# Isolation of Extracellular Vesicles From the Bronchoalveolar Lavage Fluid of Healthy and Asthmatic Horses

**DOI:** 10.3389/fvets.2022.894189

**Published:** 2022-06-21

**Authors:** Nina Höglund, Ninna Koho, Heini Rossi, Jenni Karttunen, Anne-Mari Mustonen, Petteri Nieminen, Kirsi Rilla, Sanna Oikari, Anna Mykkänen

**Affiliations:** ^1^Department of Equine and Small Animal Medicine, Faculty of Veterinary Medicine, University of Helsinki, Helsinki, Finland; ^2^Institute of Biomedicine, School of Medicine, Faculty of Health Sciences, University of Eastern Finland, Kuopio, Finland; ^3^Department of Environmental and Biological Sciences, Faculty of Science and Forestry, University of Eastern Finland, Joensuu, Finland

**Keywords:** airway disease, bronchoalveolar lavage fluid (BALF), equine asthma, extracellular vesicle (EV), inflammation, size-exclusion chromatography

## Abstract

Extracellular vesicles (EVs) are membrane-bound particles that engage in inflammatory reactions by mediating cell–cell interactions. Previously, EVs have been isolated from the bronchoalveolar lavage fluid (BALF) of humans and rodents. The aim of this study was to investigate the number and size distribution of EVs in the BALF of asthmatic horses (EA, *n* = 35) and healthy horses (*n* = 19). Saline was injected during bronchoscopy to the right lung followed by manual aspiration. The retrieved BALF was centrifuged twice to remove cells and biological debris. The supernatant was concentrated and EVs were isolated using size-exclusion chromatography. Sample fractions were measured with nanoparticle tracking analysis (NTA) for particle number and size, and transmission electron microscopy and confocal laser scanning microscopy were used to visualize EVs. The described method was able to isolate and preserve EVs. The mean EV size was 247 ± 35 nm (SD) in the EA horses and 261 ± 47 nm in the controls by NTA. The mean concentration of EVs was 1.38 × 10^12^ ± 1.42 × 10^12^ particles/mL in the EA horses and 1.33 × 10^12^ ± 1.07 × 10^12^ particles/mL in the controls with no statistically significant differences between the groups. With Western blotting and microscopy, these particles were documented to associate with EV protein markers (CD63, TSG101, HSP70, EMMPRIN, and actin) and hyaluronan. Equine BALF is rich in EVs of various sizes, and the described protocol is usable for isolating EVs. In the future, the role of EVs can be studied in horses with airway inflammation.

## Introduction

Equine asthma (EA) is a common inflammatory disease of the lower airways. The equine condition resembles human non-allergic asthma in many ways, and horses can, thus, provide a naturally occurring translational model for asthma (1). Extracellular vesicles (EVs) are bioactive particles that have recently been associated with many pathological conditions. The number of EVs isolated from bronchoalveolar lavage fluid (BALF) increases in human asthma, and the potential role of EVs as drivers of inflammation in asthmatic airways provides an interesting research premise (2, 3). Better understanding of the role of EVs in asthma pathogenesis may reveal new diagnostic possibilities and novel treatment options to alleviate inflammation and to prevent remodeling of the airways.

EA is caused by an immunological reaction triggered by external factors, such as airborne antigens. Hyperresponsive airways give rise to inflammation of the bronchi, excess airway mucus accumulation, airway wall remodeling, and bronchoconstriction (4). EA can be classified as mild/moderate or severe. Horses suffering from mild to moderate asthma often have poor performance but do not necessarily show overt clinical signs, such as cough and respiratory distress, which are present in the severe form. Currently, the asthma diagnosis is obtained through physical examination, blood analysis, bronchoscopy, and airway cytology, with BALF collection remaining the standard sampling method for lower airway diagnostics. However, all horses with clinical signs do not show marked alterations in inflammatory cell counts in their airway samples, or abnormalities in physical examination and airway endoscopy, and the correlation between parameters varies (5).

EVs are phospholipid bilayer-covered particles that are released from cells to the local environment and circulation (6). They are mainly categorized into three groups: exosomes, microvesicles, and apoptotic bodies. However, the groups overlap in size with each other, and the precise morphological identification, labeling, and isolation of EVs are challenging. EVs are released from multivesicular bodies or by outward budding from the plasma membrane, and they are associated with proteins derived from the lipid bilayer and the cytosol (7, 8). They play a crucial role in cell–cell communication and organization of the extracellular matrix and are present in all body fluids (6). EVs carry diverse cargos that include nucleic acids, proteins, lipids, and signaling molecules, and they can induce both protective and pathologic effects (9). EV communication occurs through EV surface receptors and endocytosis by the cells or by fusion of the EV and cell membranes (6). In a recent study by Nirujogi et al. (2022) on mice BALF, acute lung injury increased the release of EVs (10). In asthma, EVs are mostly recognized to promote remodeling and to disseminate inflammation by upregulating and activating proinflammatory responses, however, there are also research data supporting anti-inflammatory activity of EVs (11, 12). Additionally, EVs originate from different cells, such as eosinophils, mast cells, and neutrophils, according to disease type (10, 12). EVs have potential as biomarkers for diseases and as therapeutic agents due to their proinflammatory and immune system triggering properties (13–15).

Among bioactive molecules transported by EVs is hyaluronan (HA) (16), a non-sulfated glycosaminoglycan that is a constituent of the extracellular matrix participating in cell proliferation, inflammatory processes, and in the regulation of fluid balance in the interstitium (17, 18). High-molecular-weight HA is considered to have anti-inflammatory action, while low-molecular-weight HA can induce proinflammatory effects (19). Earlier data have shown increased amounts of lower-molecular-weight HA fragments in airways of asthma patients that could contribute to chronic inflammation and remodeling of airways (20).

The complexity of biological fluids and the nature of EVs pose the main challenges to their isolation. Therefore, a combination of several methods is necessary for successful EV extraction (13, 21). Gel chromatography, or size-exclusion chromatography (SEC), has become a popular method for EV extraction. It is based on the technique where a resin-packed column, which does not absorb or react with the fluid, separates molecules according to size. Molecules smaller than the determined size enter resin matrix pores, which results in the delayed passage of smaller particles through the column. The technique cleans soluble non-EV proteins from the sample but preserves EV functionality (13, 22). Nanoparticle tracking analysis (NTA) is a widely used method to determine the size and concentration of small particles, and transmission electron microscopy (TEM) and confocal laser scanning microscopy (CLSM) can be used to directly visualize EVs (23–25). In horses, EVs have been successfully isolated from plasma, synovial fluid, uterus, bone marrow, and adipose tissue-derived cells (26–31). However, EVs have not been previously isolated from equine BALF, and the types and content of EVs produced in equine lungs remain unknown.

We aimed to isolate EVs from equine BALF and to investigate their numbers, size distribution, and content in horses suffering from naturally occurring asthma. We hypothesized that (i) EA horses would have increased levels of EVs compared to controls, (ii) EV size distribution would differ between EA horses and healthy horses, and (iii) BALF EVs would transport HA with potential to affect airway inflammation. This study provides new insight to the role of EVs in the pathophysiology of EA and an additional method for EA diagnostics and research. This knowledge may be applicable to other species and contribute to translational asthma research.

## Materials and Methods

### Sample Collection

This was a prospective clinical case-control study conducted at the Department of Equine and Small Animal Medicine, Faculty of Veterinary Medicine, University of Helsinki. The Project Authorization Board in the Regional State Administrative Agency accepted the experimental animal license (ESAVI/3285/2020), and horse owners provided informed consent to allow the participation of their animals in the study.

A total of 54 privately-owned adult horses or ponies were recruited for the study, 35 of which had chronic or recurrent signs of asthma (cough >4 months at rest or when exercised, bilateral nasal discharge, poor performance, abdominal breathing pattern) and 19 were clinically healthy controls. Inclusion criteria were an age >5 years, no signs of infection during the previous 2 months, and no medication during the past month. Exclusion criteria included findings in blood analyzes or examinations suggestive of infection or other respiratory tract diseases, such as laryngeal dysfunction, arytenoid chondropathy, infectious bronchopneumonia, interstitial pneumonia, or neoplasia. The control horses were preferably recruited from the same stables as the asthmatic horses to minimize any environmental bias caused by the stabling conditions.

The examination and sample collection procedures included a physical examination and blood sampling for hematology and biochemistry, after which the horses were sedated intravenously with a combination of detomidine (0.01 mg/kg, Domosedan, Orion, Espoo, Finland) and butorphanol (0.005–0.01 mg/kg, Butordol, Intervet, Boxmeer, the Netherlands) for airway endoscopy and bronchoalveolar lavage. A video endoscope (Pentax EC3870, Tokyo, Japan, length 170 cm, diameter 11 mm) was used to visualize the airways and to transendoscopically collect BALF. Prior to BALF sampling, the airways were locally anesthetized with 1% lidocaine solution (40 mL for horses and 20 mL for ponies, Lidocain, Orion). BALF collection was performed with sterile 0.9% saline (360 mL for horses and 240 mL for ponies) injected in one aliquot and manually aspirated from the right lung with 40–70% of the injected volume retrieved. The BALF samples were placed on ice and processed within 60 min of collection.

### BALF Cytology, EV Isolation, and Visualization

The BALF sample syringes of each horse were pooled, 20 mL was stored at −20°C, 45 mL was prepared for EV isolation, and the remaining fluid was cytocentrifuged and stained with May–Grünwald–Giemsa stain for the differential counts of inflammatory cells performed by an experienced investigator by counting 300 cells. For EV isolation, BALF was centrifuged using polypropylene Falcon centrifuge tubes (Thermo Fisher Scientific, Waltham, MA, USA) at 4°C, first at 300 × *g* for 10 min followed by supernatant centrifugation at 3000 × *g* for 20 min to remove cells and debris. To concentrate the samples, the supernatant was centrifuged at 3800 × *g* for 90–150 min with a 10K MWCO filter (#88528, Thermo Fisher Scientific). The samples were stored at −80°C in LowBind tubes (Eppendorf, Hamburg, Germany).

EV isolation was implemented with 10 mL SEC columns with a 70 nm pore size (#ICO-70, qEVoriginal, Izon Science, Medford, MA, USA). The columns were taken to room temperature 40 min prior to use and flushed with 16 mL PBS (137 mM sodium chloride, 2.7 mM potassium chloride, and 10 mM phosphate buffer) to remove sodium azide. The samples were thawed and centrifuged at 4°C, 2000 × *g* for 10 min. First, 0.5 mL SEC fractions were collected from samples of five horses, and the particle concentration of each fraction was analyzed with NTA. The protein content of the 0.5 mL SEC fractions from three horses was measured with the bicinchoninic acid assay (#23227, Thermo Fisher Scientific) to ensure the purification of EVs. Based on the initial NTA results, the remaining samples were run through the SEC column and collected as follows: 3 mL (void), 2 mL (EV sample), 2 mL (fractions 5–8), and 5 mL (proteins). One SEC column was used for five samples. For subsequent analyses, the EV samples were concentrated to 300 μL using 10K MWCO filters (#88517, Thermo Fisher Scientific). The samples were frozen and stored at −80°C in LowBind tubes (Eppendorf).

The particles were characterized with the Nanosight LM14C (*v*3.1, Malvern Panalytical, Malvern, UK) with the camera level set at 14, and the detection threshold at 4. EVs were visualized and verified with the TEM Jeol JEM-1400 (Jeol, Tokyo, Japan) operating at 80 kV, staining was performed as described by Puhka et al. (2017) (32). TEM images were captured with the Gatan Orius SC 1000B CCD-camera (Gatan, Pleasanton, CA, USA) with 4008 × 2672 px image size and no binning. EVs were also visualized by the Zeiss Axio Observer inverted microscope equipped with the Zeiss LSM 800 confocal module (Carl Zeiss MicroImaging, Jena, Germany) as outlined previously (25). The samples were stained with CellMask Deep Red plasma membrane stain (Thermo Fisher Scientific) soluted together with Alexa Fluor 568-labeled HA binding complex (33) to double-label the EVs simultaneously with HA. In addition, CellMask Deep Red plasma membrane stain was utilized with Alexa Fluor 594-labeled phalloidin-iFluor (Abcam, Cambridge, UK) to detect cytosolic F-actin in EVs.

### Western Blotting (WB)

WB was used for protein assessment from BALF of four horses to confirm the presence of EVs. For positive controls, 0.5 μg and 2 μg of homogenized equine lung tissue (obtained from an euthanized horse donated for research purposes) was used and, for the BALF EV samples, a concentration of 1.0 × 10^13^ particles/well in sample buffer (Laemmli, Bio-Rad, Hercules, CA, USA) with 10% β-mercaptoethanol. Samples were loaded in a volume of 20 μL/well. A molecular weight ladder (Precision Plus Protein, Dual Color standard, Bio-Rad) was used. The samples were thawed and heated at 100°C for 5 min before loading to the electrophoresis gel (SDS-PAGE Mini-Protean TGX gel, Bio-Rad). Proteins were separated on gels for 40–70 min at 150–200 V in buffer (Tris/glycine/SDS, Bio-Rad) and subsequently transferred to a nitrocellulose transfer membrane (Protran 0.2 μm, Perkin Elmer, Boston, MA, USA) by running a semi-dry transfer system in 15 V for 60 min.

An antibody produced against the C-terminus of equine extracellular matrix metalloproteinase inducer (EMMPRIN, also known as CD147, neurothelin, and basigin, generated in rabbits, purified with affinity chromatography, sequence GHHVNDKDKNVRQRNAS, GenBank accession No. ABQ53583.1) was used as an inflammatory protein marker, and antibodies against anti-human cluster of differentiation 63 protein (CD63, #bs-1523R, Bioss, Boston, MA, USA), anti-human heat shock protein 70 (HSP70, #ADI-SPA-812, Stressgen Biotechnologies, Victoria, BC, Canada), and anti-human tumor susceptibility gene 101 (TSG101, #612696, BD Transduction Laboratories, San Diego, CA, USA) as positive protein markers. Anti-human translocase of outer mitochondrial membrane 20 (TOMM20, #PA5-52843, Thermo Fisher Scientific) was used as a negative protein marker and horse serum albumin antibody (#PA5-97419, Thermo Fisher Scientific) as an EV purity control. Membranes were blocked with 5% dry milk powder in Tris-Buffered Saline with Tween (TBST, 20 mM Tris, 150 mM NaCl, 0.05% Tween-20) for 60 min followed by incubation with primary antibodies with a 1:1000 dilution in 5% milk with TBST at 4°C overnight. Excess primary antibodies were washed off the membranes in TBST initially for 10 min followed by two 5-min washes.

For secondary antibodies, 1:2000 dilutions were prepared with 2.5% dry milk powder in TBST and incubated for 60 min. Secondary antibodies were polyclonal goat anti-mouse (#P0447, Agilent, Santa Clara, CA, USA) for TSG101 and polyclonal goat anti-rabbit (#P0448, Agilent) for EMMPRIN, CD63, HSP70, TOMM20, and albumin. Excess secondary antibodies were washed off the membranes in TBST for 10 min and twice for 5 min. The membranes were incubated in Chemiluminescence reagent West Atto Ultimate Sensitivity Substrate (Thermo Fisher Scientific) for 2 min and visualized using the luminescent image analyzer (LAS-3000, Fujifilm Life Science, Düsseldorf, Germany) with incremental exposure times.

### Statistical Analyses

Sample size was calculated with the number of EVs as a primary outcome, an estimated difference of three between the groups, an expected standard deviation (SD) of five, a power of 80%, and significance at <0.05. A sufficient sample size for a paired *t*-test was calculated to be 25 pairs using a previous study from a similar population of horses as a reference (34). The statistical analyses were performed using the IBM SPSS *v*25 software (IBM, Armonk, NY, USA). To compare particle concentration over the different EV sizes between the groups, the AUC (area under curve) for each horse was calculated for the interval of 50–700 nm using the trapezoid method. The effects of health status on AUC adjusted for age were determined with the analysis of covariance. The model included health status as the fixed effect and age as the continuous covariate. Normality assumptions of the analysis model were tested with residual diagnostics. A square root transformation was used for the AUC to normalize the distribution. Two outliers were detected, and as they caused skewness to the distribution, the analyses were also performed excluding the outliers. The sex distribution in the study groups was tested with the Fisher's exact test and the differences in age and body weight with the Mann–Whitney U test. The association of age to EV concentration and size was tested with the Pearson's correlation coefficient (r_p_). A *p*-value < 0.05 was considered statistically significant. The results are expressed as mean ± SD.

## Results

### Clinical Findings

The recruited horses represented several breeds (17 warmbloods, 18 coldbloods, 11 ponies, 3 standardbreds, 5 other breeds) and all sexes (41 geldings, 10 mares, 3 stallions). Out of 54 horses, 35 were included in the EA group, with owners reporting poor performance (*n* = 16), abdominal breathing pattern (*n* = 11), and nasal discharge (*n* = 9) as clinical signs in addition to cough. None of the EA horses displayed respiratory distress during clinical examinations, and there were no horses with signs of any other disease or abnormality that could potentially have caused respiratory symptoms. The control horses (*n* = 19) had no signs of respiratory disease in their medical histories or during examinations. The characteristics of the enrolled horses are described in Table 1. There were no differences in the sex ratios or body weights between the groups, but the EA horses were older than the controls (*p* = 0.01).

**Table 1:** General characteristics of enrolled asthmatic and control horses (mean ± SD).

	**Asthma (*n* = 35)**	**Control (*n* = 19)**	***P***
Age (years)	14 ± 5	10 ± 4	0.01
Body weight (kg)	492 ± 131	492 ± 106	0.83
Sex			0.68
Mare (*n*)	5 (14%)	5 (26%)	
Stallion (*n*)	2 (6%)	1 (5%)	
Gelding (*n*)	28 (80%)	13 (69%)	

### BALF Cytology, EV Isolation, and Characterization

**Figure 1:**
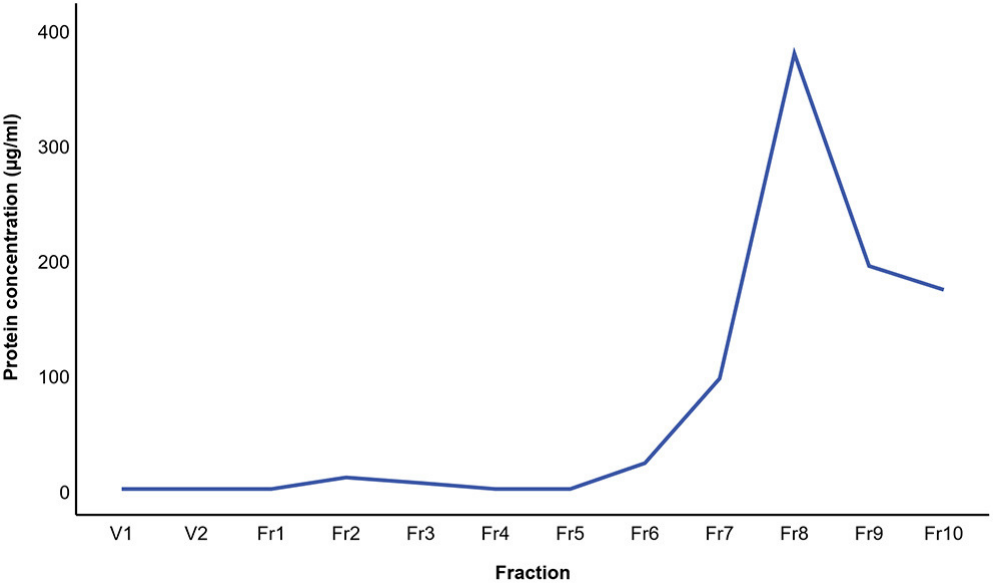
Mean protein concentration in extracellular vesicle sample fractions from three horses isolated by size-exclusion chromatography. The protein concentration was analyzed with the bicinchoninic acid assay.

**Figure 2:**
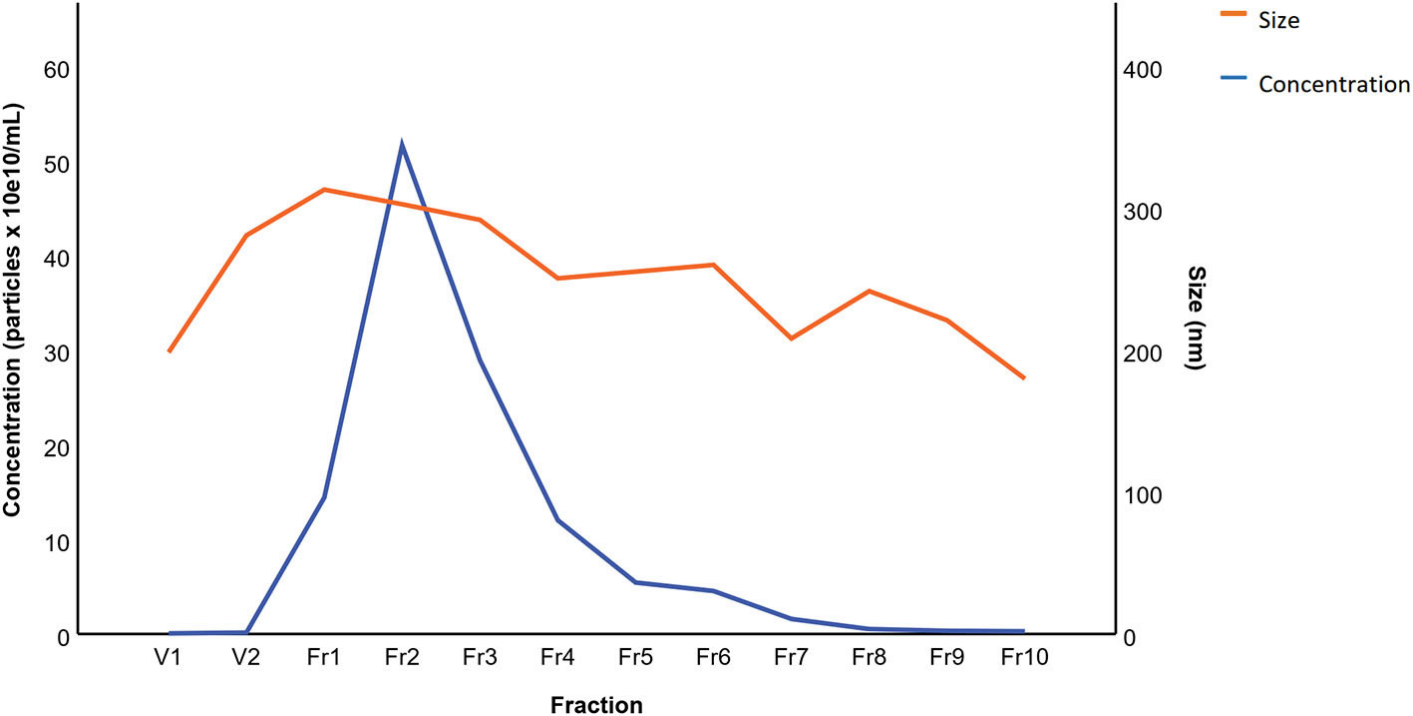
Mean particle concentration and size in extracellular vesicle sample fractions from five horses isolated by size-exclusion chromatography. The fractions were analyzed by nanoparticle tracking analysis.

**Figure 3:**
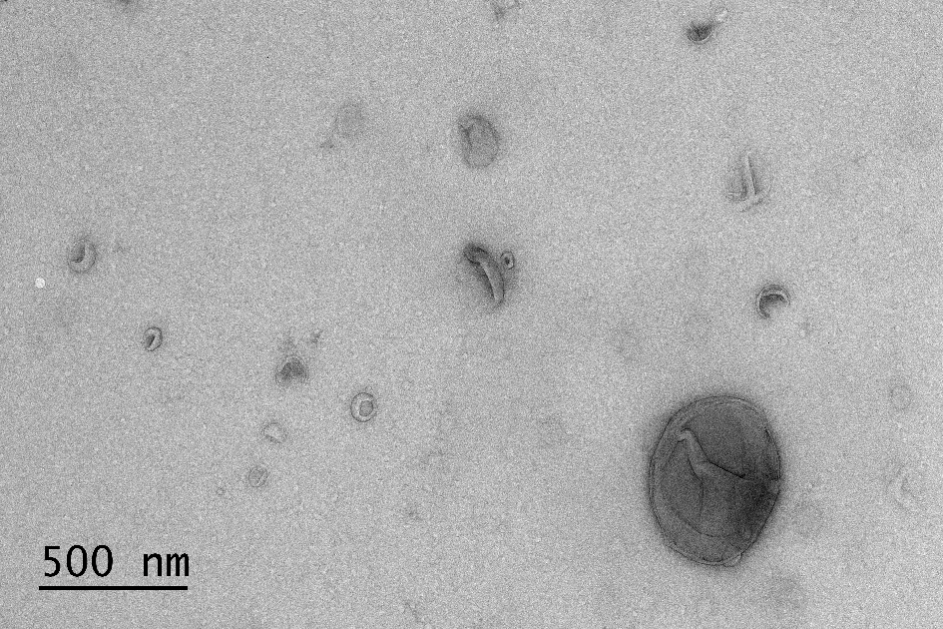
Transmission electron microscopy image of extracellular vesicles isolated by size-exclusion chromatography from the bronchoalveolar lavage fluid of an asthmatic horse.

**Figure 4:**
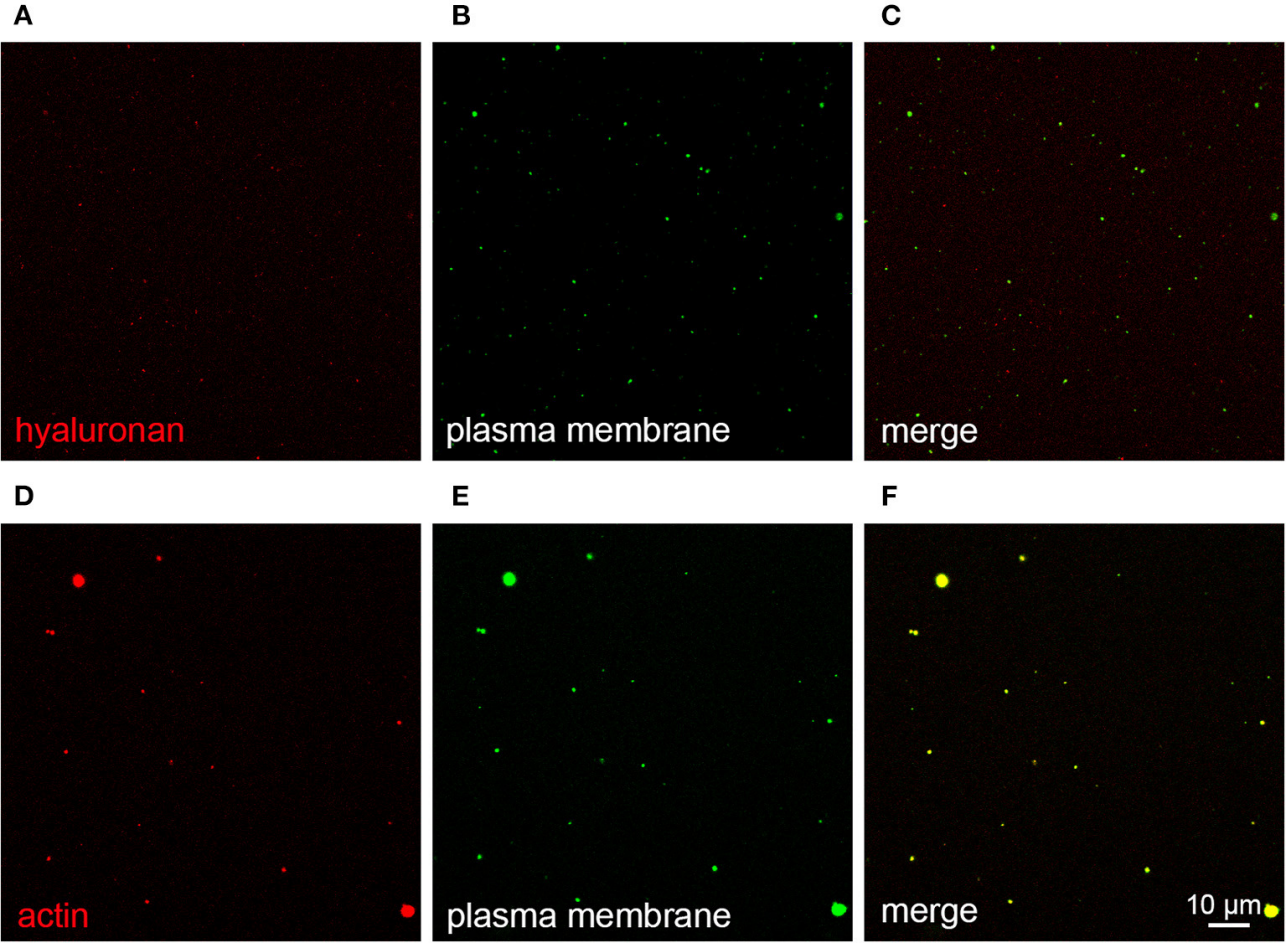
Extracellular vesicles (EVs) from bronchoalveolar lavage fluid (BALF) stained with CellMask Deep Red plasma membrane stain (pseudocolored green, **B,E**), combined with Alexa Fluor 568-labeled hyaluronan binding complex **(A)** or 594-labeled phalloidin to detect F-actin **(D)**, both pseudocolored red. Merged images are shown in **C,F**, respectively. EVs in panels A–C were isolated by size-exclusion chromatography from BALF of a control horse. EVs in **D–F** were obtained by centrifugation and concentration of BALF of an asthmatic horse.

The results of BALF cytology are presented in Supplementary Material 1. The 0.5 mL SEC fractions were collected and analyzed with NTA from five horses. The protein measurement of the 0.5 mL SEC fractions from three horses showed a low soluble non-EV protein content (Figure 1). The highest particle concentrations were observed in fractions 1–4 (Figure 2) and, therefore, in the remaining samples these fractions were pooled into a single sample/animal with a total volume of 2 mL (EV sample) that was concentrated to 300 μL and used for further analyses. TEM imaging from pooled fractions 1–4 verified the presence of various-sized EVs up to 1000 nm, while non-EV material, such as cell debris, was also detected in the samples (Figure 3). CLSM visualized EVs that transported HA and cytosolic F-actin (Figure 4). The positive EV protein markers CD63, TSG101, HSP70, the inflammatory protein marker EMMPRIN, and the purity control marker albumin were present in the EV and lung tissue samples (Supplementary Material 2). The negative EV protein marker TOMM20 was not detected in the EV samples.

### EV Number and Size

**Figure 5:**
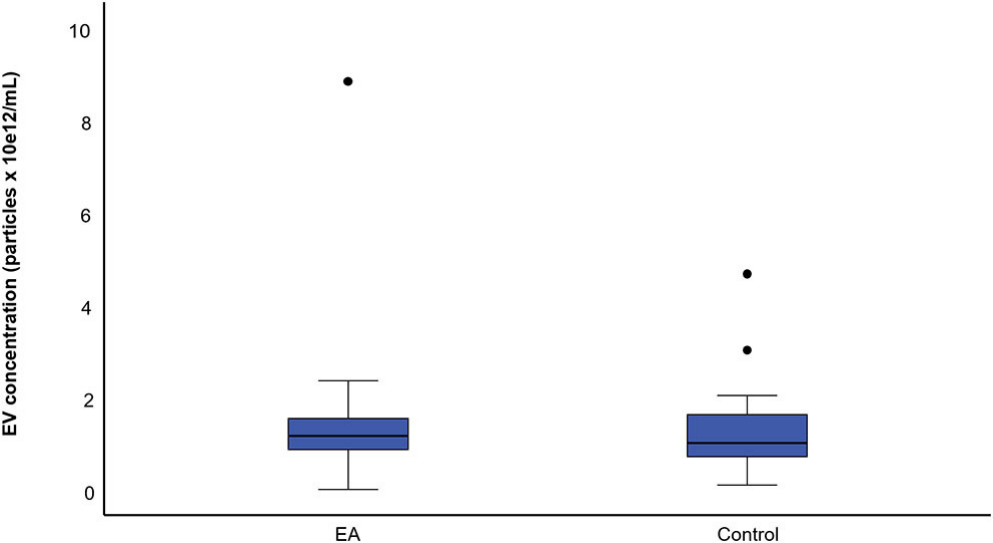
Boxplots of mean particle concentration for the asthmatic (EA) (*n* = 35) and control horses (*n* = 19). The samples were analyzed by nanoparticle tracking analysis. Each box represents the interquartile range. The horizontal line in the box represents the median, the whiskers the range, and the circles the outliers. There was no significant difference between the groups.

**Figure 6:**
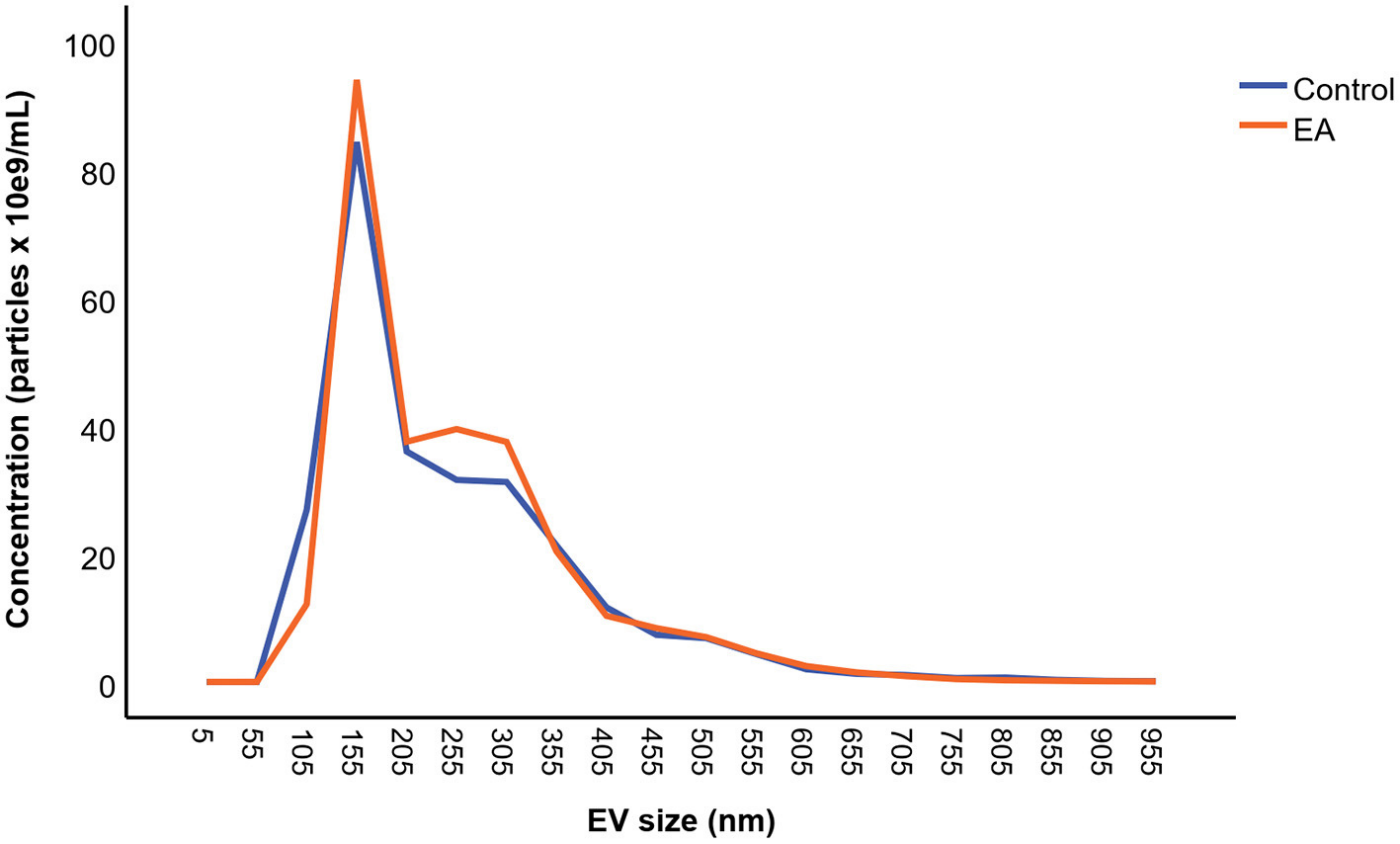
Mean particle concentration and extracellular vesicle (EV) size distribution for the asthmatic (EA) (*n* = 35) and control horses (*n* = 19). The samples were analyzed by nanoparticle tracking analysis. There was no significant difference between the groups.

The mean concentration of EVs was 1.38 × 10^12^ ± 1.42 × 10^12^ particles/mL in the EA horses and 1.33 × 10^12^ ± 1.07 × 10^12^ particles/mL in the control group determined by NTA (Figure 5). The mean size of EVs was 247 ± 35 nm in the EA horses and 261 ± 47 nm in the controls. The diameters of most EVs were between 100–350 nm (Figure 6), while TEM and CLSM visualized EV diameters up to 1,000 nm (Figure 4). There were no significant differences in the particle concentrations over the different EV sizes between the groups when tested with (*p* = 0.745) or without the outliers (*p* = 0.613). Age did not correlate significantly with the EV concentration (r_p_ = −0.194) or EV size (r_p_ = −0.154).

## Discussion

The present study describes a novel method for EV isolation from equine BALF and compares the size and particle concentration of EVs between horses with naturally occurring EA and healthy controls. The lack of a universal EV isolation protocol is a key challenge for EV research. Earlier studies have shown successful isolation of EVs from human and mouse BALF with ultrafiltration and ultracentrifugation (15, 35–37). Recently, SEC has become a popular method for isolating EVs because of the relatively pure and intact EVs that it can produce. However, the total separation of non-vesicular material from EVs remains impossible with currently available methods (35, 38, 39). Dilute samples are another potential downside of SEC, caused by the separation of the EVs into several fractions, but this can be avoided by pooling and by performing an additional concentration step. In the present study, the extraction method resulted in an EV concentration sufficient to be used in subsequent laboratory analyses, such as WB.

We analyzed the EVs isolated from BALF according to the MISEV 2018 guidelines (21) and demonstrated the presence of EVs in all main analyses. NTA and CLSM showed the presence of EV-sized particles in the samples, and TEM confirmed the presence of particles with cup-shaped morphology that is characteristic to EVs (23, 39). For WB, the MISEV 2018 guidelines (21) recommend analyzing proteins from three groups: (1). positive transmembrane protein markers, (2). positive cytosolic protein markers, and (3). negative protein markers. We detected CD63 (group 1), TSG101 (group 2), and HSP70 (group 2) from the EV preparations and lung tissue control samples. By using fluorescent phalloidin, the presence of actin (group 2) was also demonstrated in EVs with CLSM. TOMM20 (group 3) was detected by WB in 2 μg lung tissue but not in the EV samples. A plausible explanation for the absence of TOMM20 in the 0.5 μg lung tissue sample is the very low amount of the protein in equine lung tissue. TOMM20 is present in mitochondria, and an earlier study on different animal species has found that mitochondrial volume density decreases with increasing alveolar diameter (40). EMMPRIN was used as an inflammatory protein marker, as our group has previously found increased expression of matrix metalloproteinase-9 and its regulator EMMPRIN in the BALF of asthmatic horses (34). EMMPRIN, TSG101, CD63, and HSP70 proteins assessed with WB showed relatively low-intensity staining in the EV samples but high-intensity staining in the lung tissue samples (Supplementary Figure 2). This was likely caused by a low soluble protein content that is typical for samples isolated with SEC (38).

At present, there are no EV extraction methods that could purify biological fluid samples from all cell debris and proteins (35) and, in our study, TEM, CLSM, and WB analyses showed potential sample contamination. EV surface proteins are attached to the EV membrane with a smooth diffuse manner that forms a protein corona for the EV. In addition to EV proteins, other proteins and aggregates without any direct connection to EV function can attach tightly to the EV protein corona (8). This results in the co-extraction of non-EV origin proteins, which may explain the detected albumin that was used as a purity control in the EV samples. Furthermore, EV concentration measurements can be influenced by non-EV materials. The use of detergents prior to EV isolation could be a useful tactic to remove albumin from the EV samples, although EV components and subsequent analysis methods may be affected by detergent use (21, 41).

Altered concentrations of HA have been observed in several inflammatory diseases, such as asthma, rheumatoid arthritis, vascular diseases, and inflammatory bowel disease (17, 42, 43). The function of HA is dependent on its molecular weight and mechanisms of interaction, such as the access to multiple HA-binding proteins (42). We observed with CLSM that BALF EVs transported HA similar to synovial fluid EVs (25) and that the samples also contained free HA unbound to EVs. As asthma can be associated with increased secretion of HA with lower molecular size in humans (20), the potential roles of HA–EVs in its inflammatory and fibrotic processes warrant further studies to be revealed. Previously, a pretreatment with hyaluronidase led to improved recovery of EVs from equine synovial fluid with a high HA concentration (27) but, at the same time, the potentially significant physical association of EVs and HA would be lost.

Unlike in human subjects (2), the EV concentration and size did not increase in asthmatic horses. In concert with our results, the EV size distribution in humans was similar between asthmatic patients and controls (2). The particles isolated by differential centrifugation from human BALF ranged from 50 to 150 nm in size, which is less than the mean diameter in our EV samples. However, the particle count for certain-sized EVs should be interpreted cautiously because the chosen isolation method, such as the pore size in SEC, can affect the concentrations of particles by eliminating EVs of a particular diameter. As we used a pore size of 70 nm in our study, we expected that particles larger than this would leach out into the first fractions, leaving the smaller particles in the subsequent ones. The variation in the retrieved BALF volume and subsequently in the concentration of epithelial lining fluid in the samples increases sample variability, which may have an effect on the results. This can be further affected by the concentration step in the isolation protocol, and lead to large inter-individual variations observed in the NTA results.

EA prevalence varies depending on the climate and stabling conditions. Mild/moderate EA is common and reaches a prevalence of up to 66–80% (44, 45), while severe EA has a lower prevalence of 14–17% in stabled horses in the Northern hemisphere (45, 46). The milder forms have received increasing interest in recent years, as mild and moderate asthma cause economic losses worldwide due to reduced performance, which could be ameliorated through early diagnosis and treatment. The horses in our study represent a natural horse population for EA research that could also contribute to translational asthma research. However, because the symptoms were relatively mild at the time of sample collection, the low level of airway inflammation may explain why there were no statistically significant differences in the EV variables between the groups. According to a study by Moon et al. (2015) (11) on mice, the numbers of BALF EVs originating from lung epithelial cells increased in the presence of inflammation while the amount of EVs derived from other cells, such as alveolar macrophages, interstitial macrophages, or dendritic cells, remained unchanged. The clinical significance of EVs in horses with severe asthma and their relation to the extent of increase in BALF inflammatory cell counts require further studies to discover the most promising targets for translational research. Another study limitation was the age difference between the EA and control horses. However, the age did not show significant association with the concentration or size of EVs. In addition, the data from the older horses yielded clinically relevant information about the biology of asthma in this age group.

In conclusion, the results of the present study support the study hypotheses with some exceptions. Membrane-bound particles were successfully isolated from equine BALF, and the presence of positive protein markers as well as an inflammatory protein marker verified them as EVs. Moreover, EVs were observed to carry HA with potential to influence the inflammatory or fibrotic nature of asthma. However, the count and size distribution of EVs did not differ between the horses with mild/moderate asthma and the healthy controls and, therefore, the roles of EVs of various origin and cargo in airway inflammation warrant further study.

## Data Availability Statement

The original contributions presented in the study are included in the article/Supplementary Material, further inquiries can be directed to the corresponding author/s.

## Ethics Statement

The animal study was reviewed and approved by the Project Authorization Board in the Regional State Administrative Agency (ESAVI/3285/2020). Written informed consent was obtained from the owners for the participation of their animals in this study.

## Author Contributions

NH has taken part in study design, sample collection, laboratory analyses, analysis of the results, and manuscript preparation, with input from all authors. AM, HR, and NK contributed to the design and implementation of the research, to the analysis of the results, and to the writing of the manuscript. NK and JK took part in the developing of the laboratory analyses and manuscript preparation together with AM and NH. PN, A-MM, KR, and SO performed the confocal laser scanning microscopy. AM, HR, PN, A-MM, and JK were involved in the supervising of the work. All authors contributed to the article and approved the submitted version.

## Funding

Funds were received from the Finnish Veterinary Foundation, the Finnish Foundation of Veterinary Research, and the Academy of Finland (grant #322429 to PN).

## Conflict of Interest

The authors declare that the research was conducted in the absence of any commercial or financial relationships that could be construed as a potential conflict of interest.

## Publisher's Note

All claims expressed in this article are solely those of the authors and do not necessarily represent those of their affiliated organizations, or those of the publisher, the editors and the reviewers. Any product that may be evaluated in this article, or claim that may be made by its manufacturer, is not guaranteed or endorsed by the publisher.

## Supplementary Material

Supplementary Material 1 Boxplots of bronchoalveolar lavage fluid (BALF) cell percentage among the control horses (*n* = 19) and horses with equine asthma (EA; *n* = 35). Each box represents the interquartile range. The horizontal line in the box represents the median, the whiskers the range, and the black points the outliers.

Supplementary Material 2 Western blot membranes depicting the expressions of proteins EMMPRIN, CD63, HSP70, TSG101, TOMM20, and horse serum albumin in equine lung tissue and extracellular vesicles from asthmatic (EV1) and control (EV2) bronchoalveolar lavage fluids.

## Abbreviations

AUC: Area under curve
BALF: Bronchoalveolar lavage fluid
CD63: Cluster of differentiation 63
CLSM: Confocal laser scanning microscopy
EA: Equine asthma
EMMPRIN: Extracellular matrix metalloproteinase inducer
EV: Extracellular vesicle
HA: Hyaluronan
HSP70: Heat shock protein 70
NTA: Nanoparticle tracking analysis
r_p_: Pearson's correlation coefficient
SD: Standard deviation
SEC: Size-exclusion chromatography
TBST: Tris buffered saline solution with 0.1% Tween
TEM: Transmission electron microscopy
TOMM20: Translocase of outer mitochondrial membrane 20
TSG101: Tumor susceptibility gene 101
WB: Western blotting.
