# Supplementary figures and images for: Isolation of Extracellular Vesicles From the Bronchoalveolar Lavage Fluid of Healthy and Asthmatic Horses
Source: Front Vet Sci. 2022 Jun 21;9:894189. doi: 10.3389/fvets.2022.894189 (PMC9255554; doi:10.3389/fvets.2022.894189)

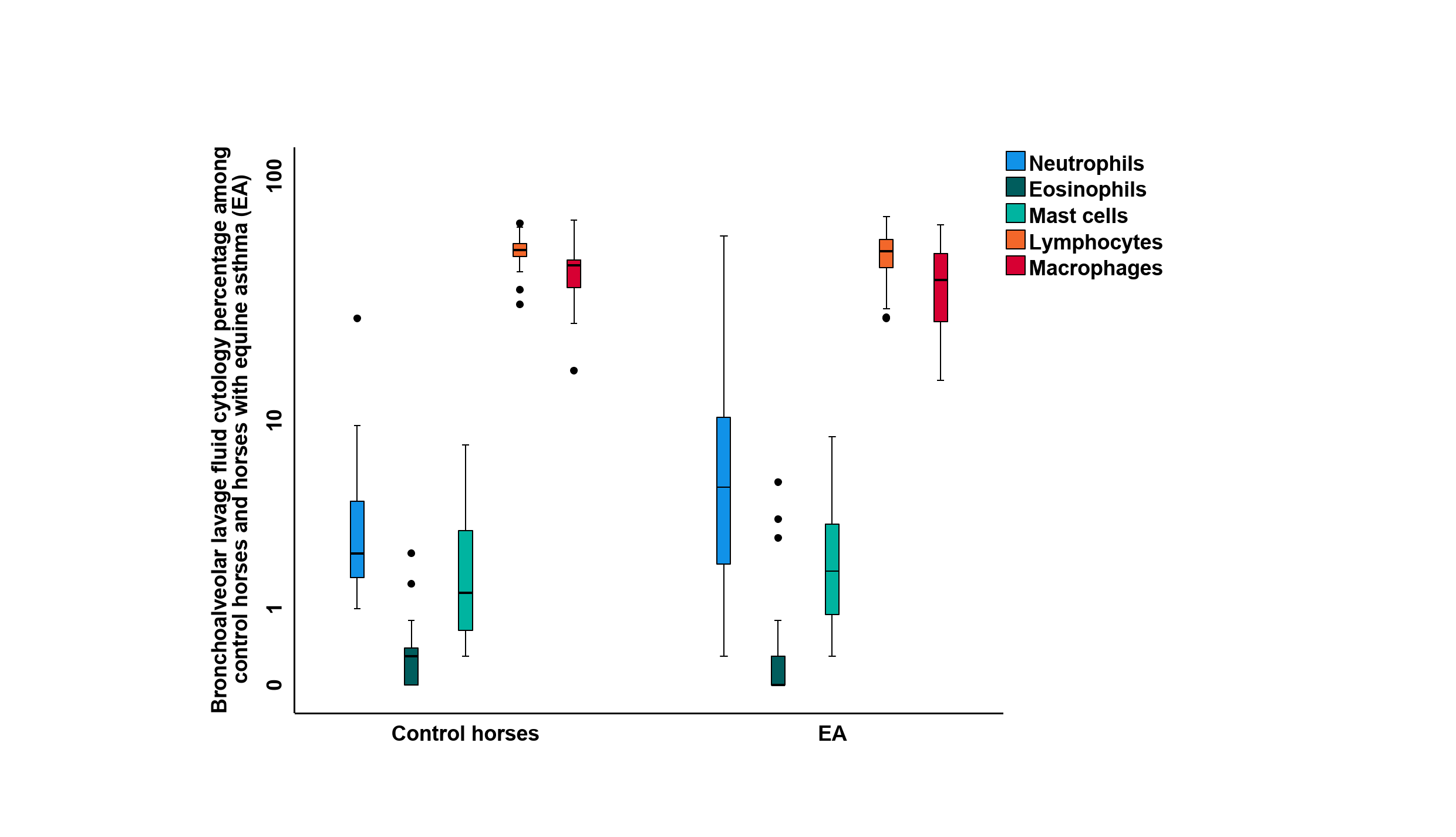

Supplement: Supplementary Material 1 — Boxplots of bronchoalveolar lavage fluid (BALF) cell percentage among the control horses (n = 19) and horses with equine asthma (EA; n = 35). Each box represents the interquartile range. The horizontal line in the box represents the median, the whiskers the range, and the black points the outliers. [file Image_1.TIF]

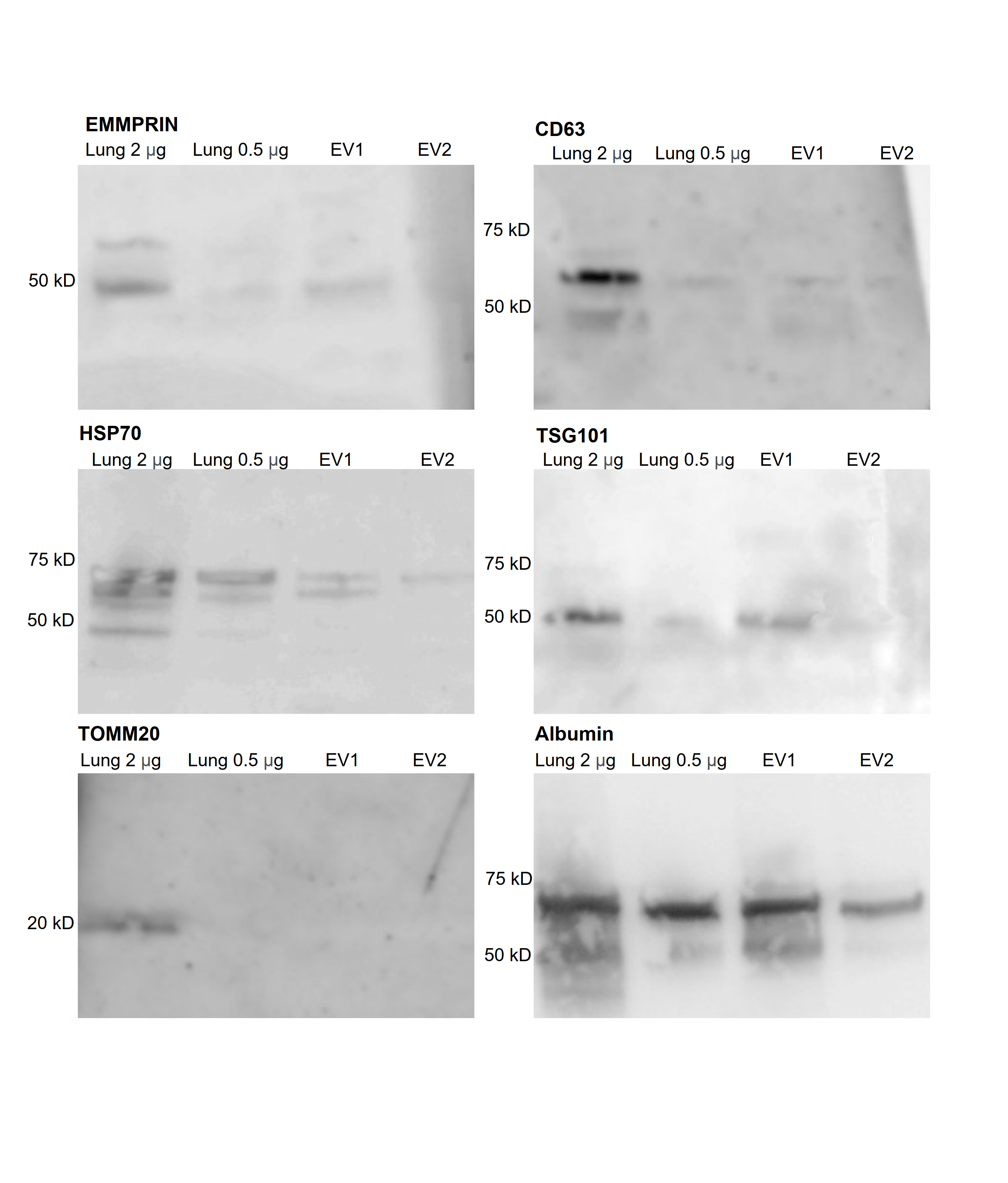

Supplement: Supplementary Material 2 — Western blot membranes depicting the expressions of proteins EMMPRIN, CD63, HSP70, TSG101, TOMM20, and horse serum albumin in equine lung tissue and extracellular vesicles from asthmatic (EV1) and control (EV2) bronchoalveolar lavage fluids. [file Image_2.TIF]
